# Comparison of the histology of the skin of the Windsnyer, Kolbroek and Large White pigs

**DOI:** 10.4102/jsava.v89i0.1569

**Published:** 2018-09-25

**Authors:** Davison Moyo, Monica Gomes, Kennedy H. Erlwanger

**Affiliations:** 1 School of Physiology, University of the Witwatersrand, South Africa

## Abstract

The skin is a protective barrier, and an endocrine, sensory and thermoregulatory organ. We investigated whether the skin of local pigs had beneficial anatomical traits compared to exotic pigs to withstand the increased heat loads predicted under future climate change scenarios. Full-thickness skin specimens were obtained from the dorsal interscapular, lateral thoraco-abdominal and ventral abdominal regions of intact boars (age 6–8 months) of two local breeds of pigs (Windsnyer [*n* = 5] and Kolbroek [*n* = 4]) and an exotic pig breed (Large White [*n* = 7]). The skin sections were stained with a one-step Mallory–Heidenhain stain and Fontana stain (melanin). Sweat gland perimeter was measured using Image J software. The Windsnyer breed had the thinnest dermis layer while the Large White had the thickest dermis layer across all the three body regions (analysis of variance [ANOVA]; *p* < 0.001). The Windsnyers had widely spaced dermal pegs compared to the other breeds. The Windsnyers had significantly more superficial and larger (~1 mm depth; 4.4 mm perimeter) sweat glands than the Kolbroek (~3 mm depth; 2.2 mm perimeter) and Large White (~4 mm depth; 2.0 mm perimeter) pigs (ANOVA; *p* < 0.001). The Windsnyers had visibly more melanin in the basal layer, the Kolbroek pigs had very little and the Large Whites had none. The functionality of the sweat glands of the Windsnyer breed needs to be established. The skin from the Windsnyer breed possesses traits that may confer a protective advantage for the increased solar radiation and ambient temperatures predicted with climate change.

## Introduction

The skin, which is an interface between an animal and its environment (Costin & Hearing 2007), has several important functions that include protection, homeostasis and sensation. The skin provides a physical barrier that protects underlying tissues from physical abrasion, bacterial invasion, dehydration and ultraviolet (UV) light (Costin & Hearing 2007). It also contributes to controlling the loss of water and solutes from the body (Meyer et al. 2011; Z˙ak et al. 2011).

The skin is involved in the regulation of body temperature (Romanovsky 2014). It has thermoreceptors that detect thermal changes (hot and cold) (Singh et al. 2013) and then send signals to the blood vessels and sweat glands. When environmental temperatures rise above the thermoneutral zone (TNZ), the skin blood flow increases because of increased vasodilation, and thermal sweating increases in species with functional sweat glands (Singh et al. 2013). These two processes increase heat loss through the skin. When environmental temperatures fall below the TNZ, increased vasoconstriction and reduced sweat production occur to conserve heat. The thickness of the skin layers (particularly subcutaneous fat), body weight, hair-coat properties, number, depth, size and functional activity of the sweat glands have an effect on the rate of heat transfer from an animal to the environment (Collier & Gebremedhin 2015).

In pigmented animals, the basal layers of the skin contain melanin. The melanin has several functions including protection of the skin, underlying tissues and organs against UV-induced skin damage through its optical and chemical filtering properties (Costin & Hearing 2007). The traits of the skin may play an important role in the capacity of animals to adapt to the increased thermal loads because of climate change.

Globally, climate change is predicted to result in spatial increases or decreases in surface temperatures and high solar radiation in the summer months. Based on the currently available data, the average world surface temperature increased by 0.8 °C (0.72 °C – 0.85 °C] when comparing the 1850–1900 and the 2003–2012 periods (Intergovernmental Panel on Climate Change 2013). South Africa lies partly in the tropics and already seems to be experiencing an increase in annual temperatures. It is reported that the average annual temperatures in South Africa have increased by at least twice the observed global average of 0.8 °C per decade reported in the Intergovernmental Panel on Climate Change (IPCC) Assessment Report No. 4 between 1960 and 2010 (Department of Environmental Affairs (DEA) 2013). Solar radiation considerably increases the environmental thermal load on animals during summer months (Spiers 2012). Studies on skin characteristics demonstrated that indigenous breeds of cattle (*Bos indicus*) in Australia (Pan 1962), sheep in Brazil (McManus et al. 2011) and the indigenous Creole pigs in the Caribbean (Renaudeau, Leclerck-Smekens & Herin 2006) tolerated heat stress better than the exotic breeds of livestock. It is not known whether the local pigs in South Africa also possess traits that may give them an advantage to cope with the high predicted thermal loads.

Pig production is important to the livelihoods of subsistence farmers in South Africa where the pigs contribute to nutrition (protein), food security, poverty alleviation, enhanced livelihood and employment creation for the rural communities (Antwi & Seahlodi 2011; Dietze 2011; Mergenthaler, Weinberger & Qaim 2009). The subsistence farmers mainly use two local breeds of pig, the Windsnyer and the Kolbroek (Halimani et al. 2010). The commercial pig farming sector relies mainly on exotic breeds such as the Large White and Landrace.

Despite the roles of the skin in thermoregulation and as a protective barrier, there is limited information on the characteristics of the skin of local pigs in southern Africa. Madzimure et al. (2012) in a study on the gross morphometry of the skin of Windsnyer and Large White pigs found that the local Windsnyer had longer hair, greater hair density and a thicker fat layer than the Large White. The study by Madzimure et al. (2012) was limited in that it did not investigate the histological structure of the skin. Consequently, there is no information on the important morphological parameters such as thickness of the skin layers, sweat gland size and distribution as well as melanin content, all of which are associated with the physiological functions of the skin. In addition, no studies were found on the microstructure of the skin of Kolbroek pigs, which are an important livestock resource in the region.

In light of the changing climate, gaining an understanding of the anatomical traits in local pigs would allow for predictions of physiological responses of the pigs. According to Dowling (1955), if animals are adapted to a set of environmental conditions, they will have skin morphological features that will allow them to survive under a given environmental condition.

The objective of this study was to determine whether there were any differences in the thickness of the skin layers, position and size of the sweat glands, and presence of melanin of the local (Kolbroek and Windsnyer) pigs and the exotic (Large White) pig breeds in order to infer how the different breeds might be able to cope with the increased temperatures and solar radiation predicted to accompany climate change in the southern African region. As a result, this should also inform breeders about possible breeds to target in their production systems in view of climate change.

## Materials and methods

The study was a prospective interventional study that used randomly selected intact boars (Large White [*n* = 7], Windsnyer [*n* = 5] and Kolbroek [*n* = 4], 6–8 months old with a body mass of 58.8 kg ± 3.1 kg [Kolbroek], 59.6 kg ± 13.3 kg [Windsnyer] and 115.8 kg ± 18.7 kg [Large White]) to investigate the morphology of the skin. All pigs were purchased from the Agricultural Research Council (ARC) Station in Irene, Pretoria, South Africa (GPS Coordinates: -28.165430 S 28.306129 E).

All pigs were kept in the Central Animal Services Farm Animal Unit at the University of the Witwatersrand (Johannesburg). They were fed a 15% crude protein soybean or maize meal-based pig grower diet enriched with vitamins and minerals (Epol, Johannesburg, South Africa) at 2% of total body mass of the pigs in each pen for maintenance of body mass. The food was moistened with water at the ratio of 2 x feed to 1 x water. Dry wheat straw provided bedding and was changed daily before feeding. A 12:12 light–dark cycle was used throughout the experiment, with lights on at 06:00. The ambient temperature in the farm animal unit was 23.0 °C ± 2.0 °C and relative humidity was 45.0% ± 5.0%; both were recorded with a data logger (Hobo U12–013 Temp/RH/2 External Data Logger, Onset Computer Corporation, Pocasset, MA, USA).

Following sedation of the pigs by a deep intramuscular (i.m.) injection of 11 mg/kg ketamine (Bayer Animal Health Division, Isando, Johannesburg, South Africa) and 0.3 mg/kg midazolam (Roche Products, Isando, Johannesburg, South Africa), they were killed by administration of a lethal dose of sodium pentobarbitone (Euthapent, 200 mg/kg intravenously [i.v.]; Kyron Laboratories [Pty] Ltd, Benrose, Johannesburg, South Africa).

In accordance with previous protocols (Debeer et al. 2013; Renaudeau et al. 2006), full-thickness skin samples measuring approximately 20 mm × 20 mm were excised from the dorsal interscapular region, lateral thoraco-abdominal region and ventral abdominal region of the bodies of all the pigs. After excision, the skin samples were immediately fixed in 10% buffered formalin and then processed using an automatic tissue processor (Shandon Citadel 1000; Thermo Scientific, Germany). Thereafter, the samples were embedded in paraffin wax using a Tissue-Tek TEC 5 Tissue Embedding Console System (Sakura Finetek Europe B.V., Amsterdam, the Netherlands). Thin vertical sections measuring 6 *µ*m in thickness were cut using a manually operated rotary microtome (Leica RM2125RT, Leica Biosystems, Nussloch, GmbH, Germany). The sections were floated in a water bath (Electrothermal MH8504, Stachwell Sunvic Ltd, London, Great Britain) set at 50 °C and containing tissue section adhesive (Sta-On, Surgipath Medical Industries Inc, Winnipeg, Manitoba, Canada). The sections were then placed on frosted glass slides and dried on a drying plate (Slide Warmer, Precision Scientific Co, Chicago, United States) at 50 °C to dry overnight. After dewaxing and rehydration, two sections from each of the three regions were stained for 5 min in an aqueous one-step Mallory–Heidenhain stain (Cason 1950), for the evaluation of general morphology. To show the melanin in the skin, the two sections from each of the three regions were stained with Fontana stain (Renaudeau et al. 2006) for 2 h in an oven at 60 °C. After staining, the slides were washed under running tap water until the water became clear and placed in 95% alcohol for 30 s, transferred to two changes of absolute alcohol for 30 s, followed by clearing in two changes of xylene for 2 min each. The slides were then mounted with a cover slip using Entellan (Merck, KGaA, Darmstadt, Germany) as a mounting medium.

Sections stained with the one-step Mallory–Heidenhain stain were photographed under a low-power (0.75× [objective lens] and 10× [eye piece lens]) using a Nikon SMZ1500 Zoom Stereomicroscope (Nikon, Japan). The microscope was coupled with a digital colour camera (Nikon model DS-Fi1, Nikon) and a computer with board for digital capture Pixel View Play TV (1280 × 980 pixels) for image capture to allow for the measurement of the thickness of the dermis, epidermis and hypodermis. The morphometric software (Image J, NIH, http://rsb.info.nih.gov/ij/) was used to measure the thickness of the epidermis (*µ*m), dermis and hypodermis (mm). The epidermis was measured using Image J software at two different points: above the dermal papillae (the thinnest part of the epidermis) and from below the stratum corneum to the end of the rete peg (thickest part of the epidermis). Sections stained with the one-step Mallory–Heidenhain stain were also photographed under a low-power (4× [objective lens] and 10× [eye piece lens]) using a Nikon microscope. The microscope was coupled with a digital colour camera TV-Lens C-06X (Nikon) and computer with ‘board for image capture’ to allow for measurement of the perimeter and depth of the sweat glands using the morphometric software Image J. Sections that were stained with the Fontana stain were photographed under a low-power (20× [objective lens] and 10× [eye piece lens]) using a Nikon microscope for the observation of the melanin.

The thickness of the epidermis in this study is reported for the thin and thick parts of the epidermis as described above. The thickness of the layers was determined by calculating the average of four measurements of the thinnest and of the thickest parts (Table 1) (Sathar, Badlangana & Manger 2010). The height of the dermal papillae (or dermal pegs) was calculated as the difference between the thickest and thinnest parts of the epidermis. The size of the sweat glands was calculated by determining the perimeter (mm) of the sweat glands (Carvalho et al. 1995) using the Image J software. The depth of the sweat glands was determined by measuring the distance between the epidermis and the top end of the sweat glands in each body region and then four measurements per region were averaged. The amount of melanin was assigned either a negative sign (for the absence of melanin) or a positive sign (for the presence of melanin). The greater the number of positive signs, the more melanin was present.

**TABLE 1:** A comparison of the thickness of the epidermis, dermis and hypodermis of local (Kolbroek and Windsnyer pigs) and exotic Large White pigs.

Variable	Large White (*n* = 7)	Kolbroek (*n* = 4)	Windsnyer (*n* = 5)
**Thin part of epidermis (*µ*m)**			
Dorsum interscapular region	46.5 ± 11.0^a^,*	58.4 ± 7.2^b^,*	35.5 ± 3.7^c^,*
Lateral thoraco-abdominal region	43.9 ± 6.5^a^,*	55.8 ± 5.4^b^,*	48.1 ± 9.0^a,b^,*
Ventral abdominal region	36.9 ± 5.9^a^,**	41.8 ± 9.2^a^,**	36.7 ± 1.9^a^,**
**Thick part of epidermis (*µ*m)**			
Dorsum interscapular region	145.9 ± 40.6^a^,*	137.0 ± 28.1^a^,*	75.3 ± 24.0^b^,*
Lateral thoraco-abdominal region	108.5 ± 24.4^a,b^,*	139.3 ± 36.3^a^,*	79.1 ± 37.3^b^,*
Ventral abdominal region	140.1 ± 46.3^a^,*	123.6 ± 19.4^a^,*	54.8 ± 2.9^b^,*
**Height of dermal papillae (*µ*m)**			
Dorsum interscapular region	104.4 ± 39.9^a^,*	90.3 ± 34.1^a,b^,*	50.6 ± 20.3^b^,*
Lateral thoraco-abdominal region	64.7 ± 27.2^a^,*	83.0 ± 36.1^a^,*	54.2 ± 22.4^a^,*
Ventral abdominal region	103.2 ± 47.4^a^,*	80.5 ± 19.6^a^,*	23.2 ± 1.8^b^,**
**Dermis (mm)**			
Dorsum interscapular region	4.5 ± 1.0^a^,*	3.3 ± 0.2^b^,*	1.1 ± 0.1^c^,*
Lateral thoraco-abdominal region	5.4 ± 1.3^a^,*	3.3 ± 0.5^b^,*	1.3 ± 0.3^c^,*
Ventral abdominal region	2.6 ± 0.5^a^,**	2.3 ± 0.3^a^,**	0.5 ± 0.1^b^,**
**Hypodermis (mm)**			
Dorsum interscapular region	8.3 ± 2.3^a^,*	10.4 ± 4.7^a^,*	1.5 ± 0.2^b^,*
Lateral thoraco-abdominal region	10.5 ± 3.3^a^,*	8.6 ± 5.2^a^,*	1.3 ± 0.4^b^,*
Ventral abdominal region	4.4 ± 1.7^a,b^,**	5.4 ± 0.5^a^,**	1.1 ± 0.5^b^,**

Note: Data are presented as mean ± standard deviation.

a, b, c Values within a row with different superscripts differ significantly at *p* < 0.05 between the breeds.

*, ** Values within a column with different superscripts differ significantly at *p* < 0.05 between the body regions.

Statistical analyses were performed using GraphPad Prism 6 (GraphPad Software, Inc., La Jolla, CA, United States). The data were analysed using repeated measures two-way analysis of variance (ANOVA) at 5% level. The thickness of the epidermis, dermis, hypodermis, height of the dermal papillae (or dermal pegs), size (perimeter) and depth of the sweat glands were compared between breeds across body regions. When the ANOVA revealed significant differences between the means, the Tukey’s multiple comparison *post-hoc* test was performed to detect the differences in the anatomical traits between the breeds and between the three body regions within a breed. Values are reported as mean ± SD. *p* < 0.05 was considered statistically significant.

### Ethical consideration

All experimental procedures used in this study were approved by the Animal Ethics Screening Committee (AESC) of the University of the Witwatersrand, Johannesburg, South Africa (AESC Clearance Certificate Number 2010/58/04). The current study was part of a bigger project on thermoregulation and febrile responses in pigs (data not presented in this report).

## Results

### Epidermis

The thin part of the epidermis was significantly different (*F*_2,13_ = 7.31; *p* = 0.0075) among the breeds, with the Kolbroek having a significantly thicker epidermis than the Large White (*p* < 0.01) and the Windsnyer (*p* < 0.001). There were no significant differences (*p* > 0.05) in the thickness of the thin part of the epidermis between the Large White and Windsnyer. When considered by specific region, in the dorsal interscapular region, the Windsnyer had a significantly different thin part compared to the Large White (*p* < 0.001) and the Kolbroek (*p* < 0.01) with the Kolbroek having a significantly (*p* < 0.01) thicker part compared to the Large White. In the lateral thoraco-abdominal region, the Kolbroek had a significantly thicker thick part than the Large White (*p* < 0.01), while no differences (*p* > 0.05) were detected in the thickness of the thin part among the different breeds. There were no differences in the thickness of the thin part among the breeds in the ventral abdominal region (*p* > 0.05). The thin part of the epidermis was significantly different (*F*_2,26_ = 11.08; *p* = 0.0003) in the three body regions (Table 1) within the breeds, with the ventral abdominal region having a significantly thinner epidermis than the dorsal interscapular (*p* = 0.002) and lateral thoraco-abdominal (*p* = 0.0001) regions. The thin part of the epidermis was not significantly different (*p* = 0.68) between the dorsal interscapular and lateral thoraco-abdominal regions. There was a significant correlation between the breed of pig and body region (*F*_4,26_ = 3.19; *p* = 0.030). The differences among breeds were not consistent across body regions. The epidermis of the Windsnyer was particularly thin on the dorsal interscapular, while in the Large White and Kolbroek, it was thin in the ventral abdominal region.

The thick part of the epidermis was significantly different (*F*_2,13_ = 15.34; *p* = 0.0004) among the breeds with the Windsnyer having a significantly thinner part than the Large White (*p* < 0.0001) and Kolbroek (*p* < 0.001). The thickness of the thick part of the epidermis was not significantly different (*p* > 0.05) between the Large White and Kolbroek. In the dorsal interscapular region, there was no significant difference in the thickness between the Large White and Kolbroek (*p* > 0.05). However, the thick part in the Windsnyer was significantly thinner than in the Large White (*p* < 0.001) and the Kolbroek (*p* < 0.01). In the lateral thoraco- abdominal region, there were no differences in the thickness of the thick part of the Large White compared to the Kolbroek (*p* > 0.05) and Windsnyer (*p* > 0.05). However, the Windsnyer pigs’ thick part of the epidermis was significantly less thick than that of the Kolbroek pigs (*p* < 0.01). In the ventral abdominal region, the thick part of the epidermis of the Windsnyer was significantly less thick compared to the Large White (*p* < 0.0001) and the Kolbroek (*p* < 0.001), while there was no significant difference in the thickness between the Large White and Kolbroek (*p* > 0.05). There were no significant differences (*F*_2,26_ = 0.78; *p* = 0.47) in the thickness of the thick part of the epidermis across the three body regions. There was no significant correlation between the breed of pig and body region (*F*_4,26_ = 1.58; *p* = 0.21).

**FIGURE 1:**
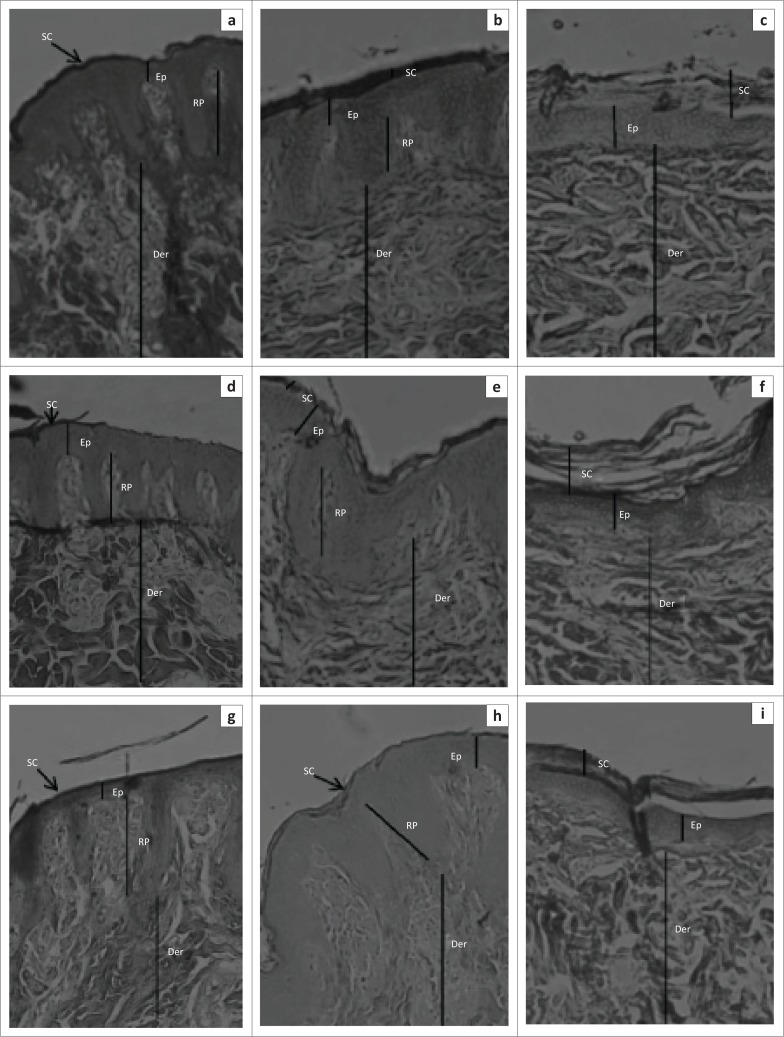
Representative skin sections from the (a, b, c) dorsal interscapular region (d, e, f), lateral thoraco-abdominal region and (g, h, i) ventral abdominal region of (a, d, g) Windsnyer (b, e, h), Kolbroek and (c, f, g) Large White pigs. The three sections from the different body regions of the three breeds of pigs show the epidermis (Ep), dermis (Der) and stratum corneum (SC) and rete pegs (RP) (Mallory–Heidenhain stain; objective: 10×).

The height of the dermal papillae was significantly different (*F*_2,13_ = 9.98; *p* = 0.0024) among breeds, with the Windsnyer having significantly shorter dermal papillae than the Large White (*p* < 0.001) and Kolbroek (*p* < 0.01). The height of the dermal papillae was not significantly different (*p* > 0.05) between the Large White and Kolbroek. In the dorsal interscapular region, the Windsnyer had significantly (*p* < 0.01) shorter dermal papillae than the Large White. There were no significant differences in the height of the dermal papillae between the Kolbroek and the Large White (*p* > 0.05) and between the Kolbroek and Windsnyer (*p* > 0.05). In the lateral thoraco-abdominal region, there were no significant (*p* > 0.05) differences in the height of the dermal papillae among the three breeds of pigs. In the ventral abdominal region, the Windsnyer had shorter dermal papillae than the Large White (*p* < 0.001) and the Kolbroek (*p* < 0.01), while there was no significant difference in the height of the dermal papillae between the Large White and Kolbroek (*p* > 0.05). There were no significant differences (*F*_2,26_ = 0.98; *p* = 0.39) in the height of the dermal papillae across the three regions within the breeds. There was no significant correlation between the breed of pig and body region (*F*_*4*__,26_ = 1.94; *p* = 0.13). On subjective assessment, the dermal papillae in the Windsnyer appeared widely spaced and where they occurred they were in clusters (Figure 1) compared with the Large White and Kolbroek.

### Dermis

The thickness of the dermis was significantly different (*F*_2,13_ = 59.29; *p* < 0.0001) among the breeds, with the Windsnyer having a significantly thinner dermis across the three body regions than the Kolbroek (*p* < 0.001) and the Large White (*p* < 0.0001). The Kolbroek had a thinner dermis than the Large White (*p* < 0.001) (Table 1). In the dorsal interscapular region, the Windsnyer had a significantly thinner dermis than the Large White (*p* < 0.0001) and the Kolbroek (*p* < 0.0001), while the Kolbroek had a thinner dermis than the Large White (*p <* 0.*01*). In the lateral thoraco-abdominal region, the Windsnyer also had a significantly thinner dermis than the Large White (*p* < 0.0001) and the Kolbroek (*p* < 0.001), while the Kolbroek had a thinner dermis than the Large White (*p* < 0.0001). In the ventral abdominal region, the Windsnyer had a significantly thinner dermis than the Large White (*p* < 0.0001) and Kolbroek (*p* < 0.001). However, the thickness of the dermis was not significantly (*p* > 0.05) different between the Large White and Kolbroek.

The thickness of the dermis was significantly different (*F*_2,26_ = 28.83; *p* < 0.00001) across the body regions, with the ventral abdominal region having a significantly thinner dermis (*p* = 0.0001) than the dorsal interscapular and lateral thoraco-abdominal regions (*p* = 0.0001). The thickness of the dermis was not significantly different (*p* = 0.17) between the dorsal interscapular and lateral thoraco-abdominal regions. There was a significant correlation between the breed of pig and body region (*F*_4,26_ = 5.57; *p* = 0.0022). The differences between breeds were not consistent across body regions. The dermis of the Windsnyer and Kolbroek was particularly thin on the ventral abdominal region, while in the Large White, it was thin on the dorsal interscapular and the ventral abdominal region.

### Hypodermis

The thickness of the hypodermis was significantly different (*F*_2,13_ = 17.75; *p* = 0.0002) among the breeds, with the Windsnyer having a significantly thinner hypodermis than the Kolbroek (*p <* 0.0001) and Large White (*p* < 0.0001), while there was no significant difference in the thickness of the hypodermis between the Large White and Kolbroek (*p* > 0.05) (Table 1). In the dorsal interscapular region, the Windsnyer had a significantly thinner hypodermis than the Large White (*p* < 0.001) and the Kolbroek (*p <* 0.0001). In the lateral thoraco-abdominal region, the Windsnyer had a significantly thinner hypodermis than the Large White (*p* < 0.0001) and the Kolbroek (*p <* 0.001). The thickness of the hypodermis was not significantly different (*p* > 0.005) between the Large White and Kolbroek in both the dorsal interscapular and lateral thoraco-abdominal regions. In the ventral abdominal region, the Windsnyer had a significantly thinner hypodermis than the Kolbroek (*p* < 0.01), while there was no significant difference in the thickness between the Large White and the Kolbroek (*p* > 0.05). There was also no significant difference in the thickness of the hypodermis in the ventral abdominal region of the Large White compared to the Windsnyer (*p* > 0.05).

There were significant differences (*F*_2,26_ = 14.32; *p* < 0.0001) in the thickness of the hypodermis across the body regions, with the ventral abdominal region having a significantly thinner hypodermis than the lateral thoraco-abdominal (*p* = 0.002) and dorsal interscapular (*p* = 0.001) regions. There were no significant differences (*p* = 0.94) in the thickness of the hypodermis between the dorsal interscapular and lateral thoraco-abdominal regions. There was a significant correlation between the breed of pig and body region (*F*_4,26_ = 4.78; *p* = 0.051). The differences between breeds were not consistent across body regions. The hypodermis of the Windsnyer was thin on the lateral thoraco-abdominal regions, while in the Large White and Kolbroek, it was thin in the ventral abdominal region.

### Sweat glands

**FIGURE 2:**
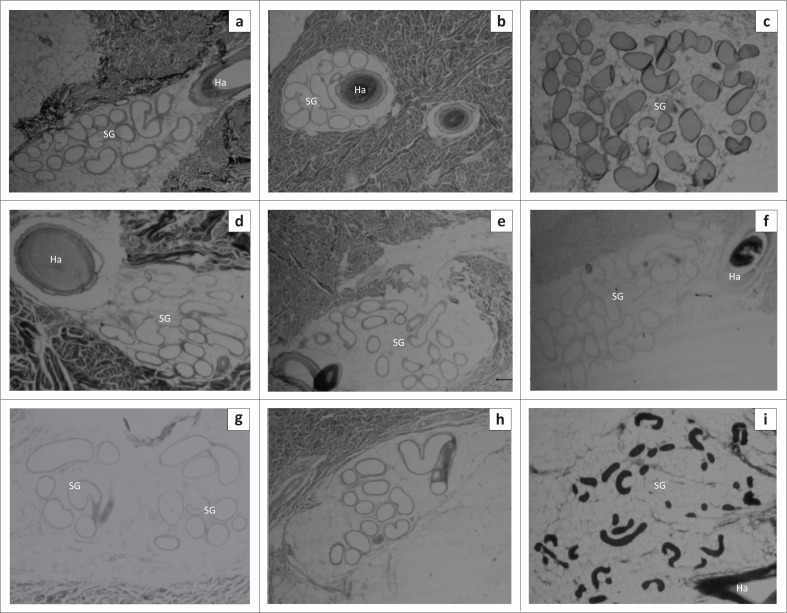
Representative skin sections from (a, b, c) the dorsal interscapular region, (d, e, f) lateral thoraco-abdominal region; and (g, h, i) ventral abdominal region of (a, d, g) Windsnyer, (b, e, h) Kolbroek and (c, f, i) Large White pigs showing the relative sizes of the sweat glands in the three body regions. (Sweat gland [SG] and hair follicle [Ha]) (Mallory–Heidenhain stain; objective: 4×).

Figure 2 shows photomicrographs indicating the relative sizes of the sweat glands from the interscapular, lateral thoraco-abdominal and the ventral abdominal regions in the different breeds of pigs. In all the regions, the sweat glands were distributed in the dermis and some sweat glands were associated with a hair follicle.

#### Sweat gland perimeter

The average perimeter (size) of the sweat glands was significantly different (*F*_2,13_ = 52.48; *p* < 0.0001) among the breeds of pigs, with the Windsnyer having a significantly larger average perimeter than in the Large White (*p* < 0.0001) and Kolbroek (*p* < 0.001) in all three body regions (Table 2). There were no significant (*p* > 0.05) differences in the perimeter of the sweat glands between the Large White and Kolbroek in all the three body regions. The average perimeter of the sweat glands was not significantly different (*p* = 0.68) between the Large White and Kolbroek. There were significant differences (*F*_2,26_ = 9.45; *p* = 0.0008) in the average perimeter of the sweat glands across the body regions with the average perimeter of the sweat glands in the ventral abdominal region being significantly smaller (*p* = 0.034) than those in the lateral thoraco-abdominal region and dorsal interscapular in all three breeds of pigs. There were no significant differences in average perimeter of the sweat glands in all the body regions (*p* = 0.94) in the Large White and Kolbroek, but the average perimeter of the sweat glands in the ventral abdominal region in the Windsnyer was significantly smaller (*p* = 0.029) compared to that in the dorsal and lateral thoraco-abdominal regions. There was no significant correlation between the breed of pig and body region (*F*_4,26_ = 2.32; *p* = 0.084).

**TABLE 2:** A comparison of the sweat gland characteristics of the local Kolbroek and Windsnyer pigs and the exotic Large White pigs.

Variable	Large White (*n* = 7)	Kolbroek (*n* = 4)	Windsnyer (*n* = 5)
**Perimeter of sweat glands (mm)**			
Dorsum interscapular region	2.1 ± 0.8^a^,*	2.1 ± 0.1^a^,*	4.7 ± 1.1^b^,*
Lateral thoraco-abdominal region	2.2 ± 0.4^a^,*	2.5 ± 0.6^a^,*	5.0 ± 0.5^b^,*
Ventral abdominal region	1.6 ± 0.4^a^,*	2.1 ± 0.2^a^,*	3.5 ± 0.7^b^,**
**Depth of sweat glands (mm)**			
Dorsum interscapular region	3.9 ± 0.9^a^,*	2.8 ± 0.7^b^,*	1.1 ± 0.2^c^,*
Lateral thoraco-abdominal region	4.0 ± 0.4^a^,*	2.9 ± 0.7^b^,*	1.0 ± 0.2^c^,*
Ventral abdominal region	3.7 ± 0.4^a^,*	2.7 ± 0.5^b^,*	1.0 ± 0.1^c^,*

Note: Data are presented as mean ± standard deviation.

a, b, c Values within a row with different superscripts differ significantly at *p* < 0.05 between the breeds.

*, ** Values within a column with different superscripts differ significantly at *p* < 0.05 between the body regions.

#### Sweat gland depth

The depth of the sweat glands was significantly different (*F*_2,13_ = 125.60; *p* < 0.0001) among the breeds of pigs, with the Windsnyer having significantly more superficial sweat glands than the Large White (*p* < 0.0001) and Kolbroek (*p* < 0.0001) in all the three body regions (Table 2). The Kolbroek had sweat glands that were more superficial (*p* < 0.001) than in the Large White in all the three body regions. There were no significant differences (*F*_2,26_ = 0.40; *p* = 0.67) in the mean depth of sweat glands within the three regions within the breeds. There was no significant correlation between the breed of pig and body region (*F*_4,26_ = 0.093; *p* =0.98).

### Distribution of melanin in the basal layers

**FIGURE 3:**
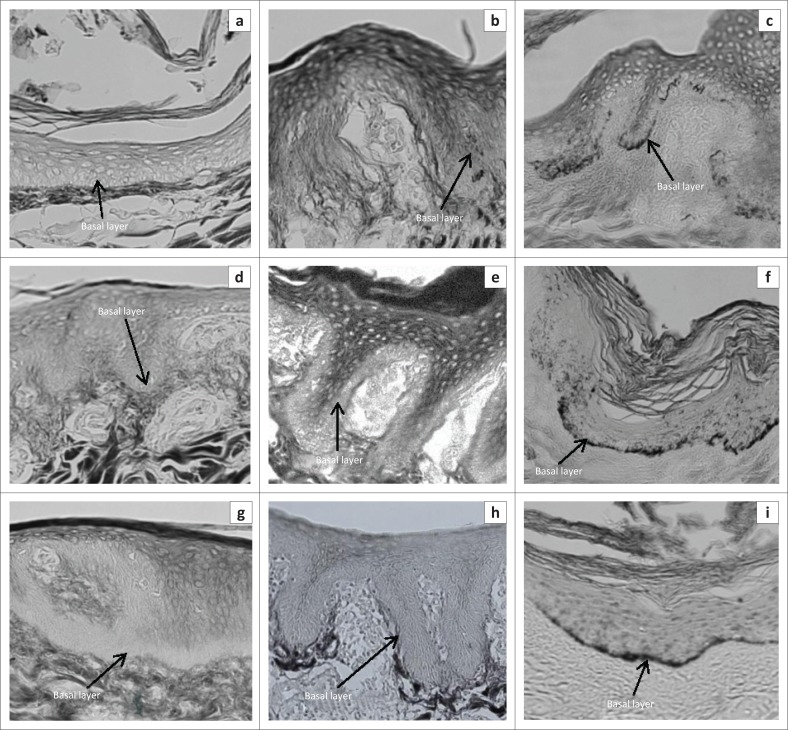
Representative skin sections (a, b, c) from the dorsal interscapular region (d, e, f), lateral thoraco-abdominal region and (g, h, i) ventral abdominal region of (a, d, g) Windsnyer (b, e, h), Kolbroek and (c, f, g) Large White pigs. The figure shows a large amount of melanin pigments in the basal layer of the epidermis in the Windsnyer pigs, very little in the interscapular region in the Kolbroek pigs and no melanin pigments in Large White pigs (Fontana staining; objective: 20×).

Figure 3 shows representative photomicrographs of sections of the three sampled regions of the epidermis showing the distribution of melanin in the basal layers. The Windsnyer had visibly more melanin in the basal layer (Table 3) than the Kolbroek, which had little melanin. The Large White pigs had no visible melanin.

**TABLE 3:** A comparison of the presence of melanin in the basal layers of the skins of the local Kolbroek and Windsnyer pigs and the exotic Large White pigs.

Variable	Large White (*n* = 7)	Kolbroek (*n* = 4)	Windsnyer (*n* = 5)
**Melanin**			
Dorsum interscapular region	−	+	++
Lateral thoraco-abdominal region	−	−	++
Ventral abdominal region	−	+	++

Note: Negative sign (−) no visible melanin present in the basal layer; positive sign (+) little melanin present in the basal layer; ++ plenty of melanin present in the basal layer.

## Discussion

The thickness of the epidermis in pigs is reportedly quite variable. For example, thicknesses of 30 *µ*m – 100 *µ*m (Morris & Hopewell 1990) and 70 *µ*m – 140 *µ*m (Meyer, Schwarz & Neurand 1978) in Yorkshire and miniature pigs, respectively, have been reported. The thickness of the epidermis in both the local Windsnyer and Kolbroek and the exotic Large White pigs in the current study ranged between 35 *µ*m and 146 *µ*m, which is comparable with the values previously noted in Yorkshire and miniature pigs. The thickness of the epidermis plays an important role in heat tolerance. Previous studies on cattle and Indian buffaloes showed that those animals with a thin epidermis were found to tolerate heat stress better than animals with a thick epidermis (Saravanakumar & Thiagarajan 1992). The Windsnyer pigs had a thinner epidermis than the Large White and Kolbroek pigs, which may confer increased heat tolerance compared to those two breeds.

The thickness of the dermis in both local pigs was less than the 3.6 mm reported for local Caribbean Creole pigs by Renaudeau et al. (2006). However, the thickness of the dermis in all the three sampled body regions of the Large White and Kolbroek pigs was within the range recorded by Andrews, Jeong and Prausnitz (2013) in Large White pigs, while the dermis in all the body regions of the Windsnyer pigs was thinner. However, the thickness of the dermis in the Large White pigs was greater in the dorsal (4.5 mm) and lateral thoraco-abdominal regions (5.4 mm) than that obtained (3.13 mm, from the ventral region) in Large White pigs by Renaudeau et al. (2006).

In all the three breeds, the dermis was thinner in the ventral abdominal region than in the other two body regions sampled. The thicker skin on the dorsal or lateral thoraco-abdominal areas is probably an anatomical adaptation to offer a physical protective barrier to trauma, as these sites are mostly likely to be exposed to the elements and potential aggressive attacks. The thickness of the dermis also plays an important role in heat tolerance of some animals, with those having a thin dermis tending to be more heat tolerant than those with a thick dermis (Wang, Suklerd & Pongthisong 2012). The Windsnyer pigs had a thinner dermis than the Large White and Kolbroek pigs in all the three body regions, suggesting that the Windsnyer pigs might have better heat tolerance than either Large White or Kolbroek pigs.

Unlike our findings, Madzimure et al. (2012) found that the Windsnyer had a thicker subcutaneous fat layer than Large White pigs. A thick hypodermis can hinder heat transfer from the body to the environment (Sokolov 1982). Differences in the thickness of the hypodermis from that of the Windsnyer pigs in the current study could be attributed to nutrition of the pigs. In their study, Madzimure et al. (2012) fed the pigs *ad libitum*, while in our study, we fed the pigs at a maintenance rate; hence, they were more likely to have a reduced fat accretion. A previous study has also shown that age and sex have an effect on fat deposition in pigs, with female pigs having a thicker fat layer than male pigs as they grew older (Bollen et al. 2004). Madzimure et al. (2012) used 3-month-old female pigs, whereas in this study, boars aged between 6 and 8 months were used. These factors (nutrition, sex and age) may explain the differences in thickness of hypodermis noted between our study and that of Madzimure et al. (2012).

The larger perimeter of the sweat glands in the Windsnyer pigs might suggest a greater capacity to lose moisture. In cattle, the sweating efficiency appears to be affected by the size, density, number and depth of sweat glands (Nay & Hayman 1956). *Bos indicus* cattle (which sweat more efficiently than *Bos taurus*) have been shown to have sweat glands that are significantly larger and more numerous than those in *Bos taurus* cattle (Pan 1962). It is important to note that most of the studies have found that sweat glands in domestic pigs are not functionally effective for thermoregulation (Ingram 1967). Sokolov (1982) stated that of the *Artiodactyla* studied, ‘the sweat glands were best developed in the wild boar’, the possible reason being that the wild boar has a thick subcutaneous fat layer that hinders heat transfer through the skin (Sokolov 1982). Given the findings in the wild boars by Sokolov (1982), it is feasible the local Windsnyer pigs might have active sweat glands, especially considering that the Windsnyer pig is reportedly closely related to the wild boar that has origins in Europe. It is important that studies on the sweat glands the Windsnyer pig be undertaken.

Melanin is important in the absorption, scattering and reflection of different wavelengths of light (Jablonski 2004). Specifically, it absorbs UV light that could damage DNA and other biological molecules. It also scavenges free radicals and regulates vitamin D3 biosynthesis by influencing the penetration of UV light through the skin. Melanin plays a role in thermoregulation and detoxification by binding to some organic molecules, drugs and heavy metals (Patel & Forsythe 2008). The amount and distribution of melanin in the skin are influenced by genetic, environmental and endocrine factors (Costin & Hearing 2007). In the current study, as expected, the Large White pigs had no visible melanin. The lack of melanin in the Large White has been attributed to a double mutation of the KIT (proto-oncogene receptor tyrosine kinase) gene, which was shown to correspond to the dominant white (I) coat colour locus on chromosome 8 (Marklund et al. 1998; Pielberg 2004).

**FIGURE 4:**
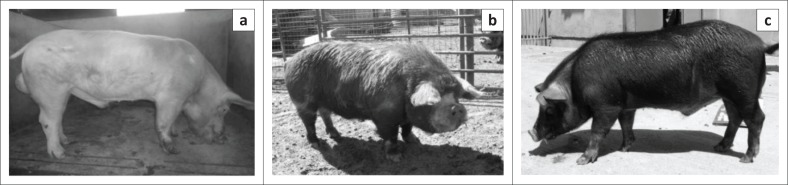
Images showing the differences in the appearance of the three breeds of pigs used in this study. (a) Large White; (b) Kolbroek and (c) Windsnyer.

A surprising observation was the presence of very little melanin in the Kolbroek pigs. From a distance, the Kolbroek pigs were observed to have black and white hair patches (Figure 4). However, close inspection of the skin showed that the Kolbroek pigs had black and white hairs, whereas the underlying skin had a uniform light-coloured appearance. The Windsnyer pigs had entirely black hairs and, like the Kolbroek, the skin underlying the hairs had a uniform light appearance superficially, but on histological examination was found to contain melanin in the basal layer of the skin. The substantial amount of melanin in the basal layer suggests that the Windsnyer pigs might have better protective, physiological and functional properties than the other breeds. As the ozone layer gets depleted, animals will have increased exposure to UV radiation, leading to increased susceptibility to its detrimental effects (de Gruijl et al. 2003).

The relatively small sample size of the local breeds is identified as a limitation to the study. In addition, the study was undertaken on only male pigs. Hormones are known to influence the anatomy of the skin; thus, there is a need to investigate the skin of female pigs as well. In an attempt to compensate for the effect of hormones and nutrition on the characteristics of the skin, the study used post-pubertal boars of a similar age. Cross-sectional sampling of different ages as well as pigs reared under different systems (e.g. free range vs penned) and different geographical regions would also have been ideal as these factors could all influence the microstructure of the skin.

## Conclusion

In South Africa, climate change is predicted to result in increased environmental temperatures that will be higher than the world average, and consequently animals in the region will experience a higher heat load than animals in other regions in the world. The Windsnyer pigs appear to possess skin anatomical traits that might buffer the effects of the changing climate such as the increased temperatures. It is thus prudent that conservation of this important genetic resource be encouraged in the region.
